# Poster Session II – Poster of Distinction II - A205 AT-HOME BREATH ANALYSIS TO UNCOVER DIETARY TRIGGERS IN IBD: A NEW FRONTIER FOR PERSONALIZED SYMPTOM MANAGEMENT

**DOI:** 10.1093/jcag/gwaf042.205

**Published:** 2026-02-13

**Authors:** A Ahmed, P Miranda, R Borojevic, J Blom, A Caminero, D Armstrong

**Affiliations:** McMaster University, Hamilton, ON, Canada; McMaster University, Hamilton, ON, Canada; McMaster University, Hamilton, ON, Canada; McMaster University, Hamilton, ON, Canada; McMaster University Faculty of Health Sciences, Hamilton, ON, Canada; McMaster University Faculty of Health Sciences, Hamilton, ON, Canada

## Abstract

**Background:**

Many patients with inflammatory bowel disease(IBD) experience gastrointestinal symptoms such as abdominal pain, bloating, diarrhea in clinical remission, which remain difficult to manage despite inactive disease. Mechanisms are unclear but likely involve microbiome alterations and fermentation of dietary carbohydrates. Undigested fermentable carbohydrates(FCHO) undergo bacterial metabolism, producing H2 and CH4, measurable via breath analysis as markers of intestinal fermentation. The AIRE-2 device(FoodMarble Inc, Dublin, Ireland), handheld breath analyzer, quantifies H2 and CH4. Integrated with smartphone app for tracking diet and symptoms, enables real-time monitoring of diet–fermentation–symptom interactions

**Aims:**

This study assessed the feasibility of using the AIRE-2 device and app to monitor diet, symptoms, and fermentation in adults with IBD in clinical remission

**Methods:**

In this prospective, single-blinded feasibility study, adults with IBD in remission completed a 5-week protocol: (1) Baseline Period(4days) of habitual diet tracking, pre- and postprandial breath testing; (2) Discovery Period(4 weeks) with 4 randomized FCHO challenges—lactose, fructose, sorbitol, inulin—each separated by 1-week washouts, preceded by a 24-hour low-FODMAP diet. Participants logged diet and symptoms via app, performed serial breath tests up to 3 hours post-challenge, completed Adult Carbohydrate Perception Questionnaire(aCPQ) pre- and post-challenge. FCHO malabsorption was a positive breath test(≥20 ppm H2 or ≥ 10 ppm CH4 from baseline); intolerance as ≥ 20-point rise in aCPQ score. Outcomes included adherence to breath, meal, challenge logs and correlations between gases and symptoms

**Results:**

Nineteen participants(12 ulcerative colitis, 7 Crohn’s disease) were enrolled. Two completed only baseline, 16 all challenges, one withdrew after two. Adherence was excellent for breath tests(78.9%), meal logging(68.4%), and challenges(84.2%). Frequent post-challenge symptoms were abdominal pain(64.7%), flatulence(41.2%), and diarrhea(41.2%). Inulin was the most common trigger, causing pain in 29.4% and bloating in 23.5%, followed by sorbitol(pain in 23.5%). Intolerance occurred in 50% for lactose, 29% fructose, and 38% each for sorbitol and inulin. Malabsorption occurred in 18.8% lactose, 35.3% fructose, and 37.5% for both sorbitol and inulin. Combined malabsorption and intolerance were seen in 18.8% lactose, 11.7% fructose, 25% sorbitol, and 12.5% inulin.

**Conclusions:**

AIRE-2 proved feasible for assessing FCHO malabsorption and intolerance in IBD remission. Findings show mismatch between symptoms and breath test results, with inulin and sorbitol as most symptom-provoking carbohydrates. At-home breath analysis may serve as non-invasive tool to identify carbohydrate sensitivities and guide personalized dietary management in IBD.

A205 Table 1: Retrospective vs. Prospective Phase Descriptive Characteristics

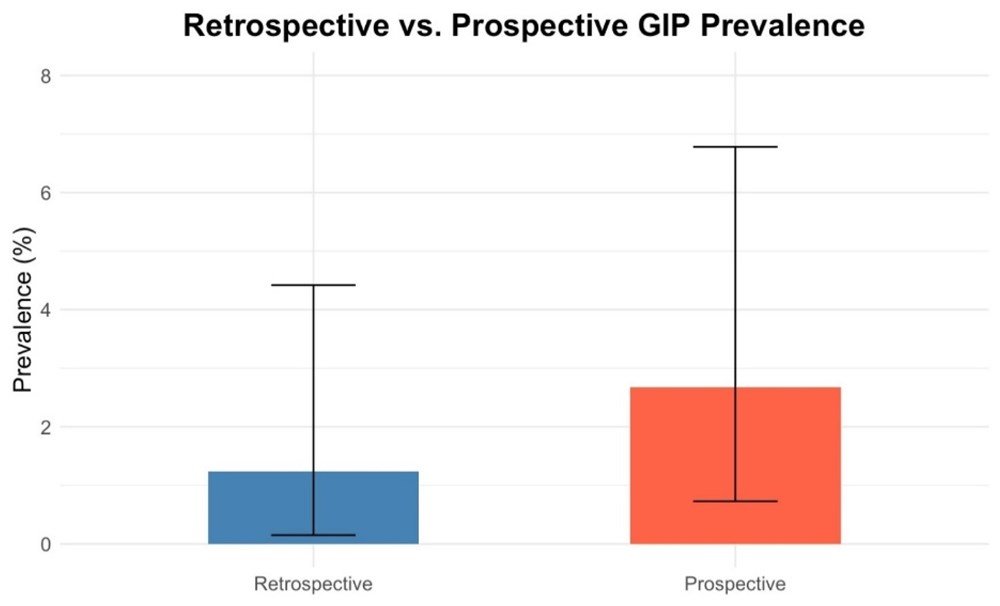

**Funding Agencies:**

Farncombe Family Digestive Health Research Institute and the Douglas Family Chair in Nutrition Research, McMaster University

**Table:** 

Phase	Retrospective	Prospective
n	161	150
Men (%)	65 (40.4)	55 (36.7)
Women (%)	96 (59.6)	95 (63.3)
Age (years, sd)	59.0 (15.5)	59.5 (16.2)
Globus (%)	2 (1.2)	5 (3.3)
Gastric Inlet Patch (%)	2 (1.2)	4 (2.6)
Dysphagia (%)	23 (14.3)	15 (10)
Gastroesophageal reflux disorder (GERD) (%)	21 (13.0)	19 (12.6)

